# Optimized Building Envelope: Lightweight Concrete with Integrated Steel Framework

**DOI:** 10.3390/ma17061278

**Published:** 2024-03-10

**Authors:** Timo Haller, Nancy Beuntner, Karl-Christian Thienel

**Affiliations:** Institut für Werkstoffe des Bauwesens, Fakultät für Bauingenieurwesen und Umweltwissenschaften, Universität der Bundeswehr München, Werner-Heisenberg-Weg 39, 85579 Neubiberg, Germany; timo.haller@unibw.de (T.H.); nancy.beuntner@unibw.de (N.B.)

**Keywords:** lightweight aggregate concrete, building envelope, integrated sheet steel framework, sustained load

## Abstract

This study presents a novel construction method for prefabricated wall elements by integrating a framework made of thin-walled sheet steel profiles into an optimized thermally insulating lightweight aggregate concrete (LAC) building envelope. The load-bearing function of the framework is provided by cold-formed Sigma-profiles, which are spot-welded to non-load-bearing U-profiles at the vertical ends. The LAC shapes the wall and stabilizes the thin-walled steel profiles against buckling, but has no further load-bearing function, thus allowing the reduction of its necessary compressive strength and subsequently minimizing its density. As a result, the LAC exhibits strength and density values well beyond existing standards, providing highly competitive thermal conductivity values that meet today’s energy requirements without the need for additional insulation materials. Tailored composite specimens verify the stabilization of load-bearing sheet steel profiles by the LAC, which not only prevents buckling but also increases the load-bearing capacity of the overall system. The feasibility of this approach is validated by the production of two prototypes, each comprising a full-sized wall, in two different precast plants using distinct process technologies.

## 1. Introduction

**Figure 1:**
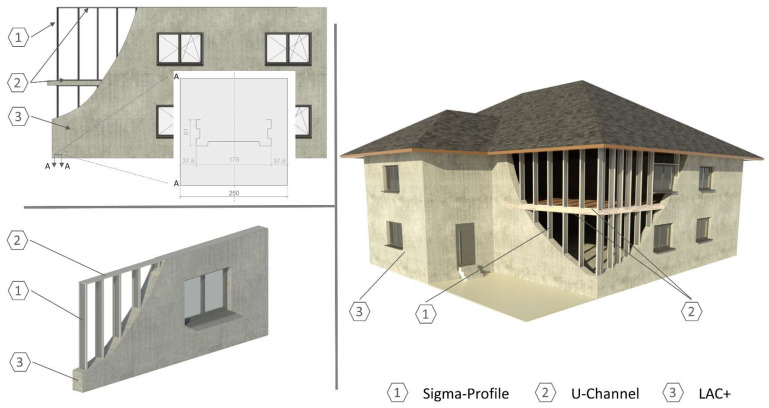
Prefabricated wall construction with sheet steel profile framework in lightweight concrete envelope. (**Top left**) Cross-section with Sigma-Profile. (**Bottom left**) Wall with LAC+ and embedded steel framework. (**Right**) CAD model of the building with new wall elements.

Concrete and steel are the most important structural building materials, which define our built environment in a variety of design forms and therefore shape our modern social lives. Intensive efforts are being made to develop new construction methods to meet increasing ecologically and economically sensitive demands. Therefore, a new prefabricated construction method for wall elements has been developed. The wall element consists of a framework made of thin-walled sheet steel profiles, which is integrated into a heat-insulating lightweight concrete building envelope. The elements can be produced in a precast concrete plant and assembled on site. Figure 1 shows a schematic representation of the new construction method.

The wall element’s primary load-bearing function is accomplished by cold-formed steel profiles. These profiles are vertically aligned in a row with defined spacing and connected at the bottom and top by spot welding to non-load-bearing U-channels. The steel profiles form a framework taking into account the subsequent wall design (e.g., technical installations, window openings, and connection elements) before being placed on the molds for concreting. The concreting is carried out with an optimized lightweight aggregate concrete (LAC). The connection of wall elements can be accomplished by means of the profile joints with mortar filling and additional mechanical joints, which are common in prefabricated construction.

The LAC developed within the scope of this work has a dry density of less than 400 kg/m^3^ and a corresponding low strength. Both are clearly below the normative limits of EN 1520 [1], but the new LAC still exhibits a great similarity in terms of material technology. For the purpose of clear differentiation, the LAC developed in the course of this work will be referred to as LAC+ in the following sections.

This new approach is designed to utilize LAC+ for its low-density characteristics, which directly influence thermal conductivity. This yields a highly competitive material in terms of energy efficiency, aligning it with increasingly stringent energy-saving regulations in the construction sector. However, as the density decreases, the compressive strength of the LAC+ also decreases. Due to its low compressive strength of around 1 N/mm^2^, the LAC+ is unsuitable for traditional load-bearing applications. At this critical point, the sheet steel framework comes into play. It provides the necessary load-bearing capacity of the building envelope, which in turn allows for an intended significant reduction in the required compressive strength of the LAC+. The sheet steel profiles are designed to efficiently utilize the material, resulting in slender profiles, which minimizes their impact on thermal conductivity. When the profiles have significant length (e.g., equal to the wall height), their failure is more likely to occur due to stability issues rather than material failure. In traditional steel construction, lateral bracing, such as cross-bracing, diagonal bracing, or shear walls, can be employed to prevent lateral deflection and ensure stability. In this case, the LAC+ provides this lateral bracing by surrounding the steel profiles and effectively preventing buckling (stability failure), ensuring that the steel profiles offer sufficient load-bearing capacity. This allows us to reduce significantly the required compressive strength of the LAC+ and opens the chance to lower the densities to such an extent that competitive thermal conductivity values unseen for LAC thus far can be achieved for meeting today’s energy requirements. The resulting connection between LAC+ and the steel framework must endure, even in the presence of time-dependent deformations, such as shrinkage and creep, or when subjected to sustained loading. This resilience is critical because damage resulting from this deformation has the potential not only to significantly reduce structural stiffness and strength but also to accelerate framework corrosion, thereby significantly impacting the overall durability [2]. The underlying fundamental concept of load transfer and construction methodology is well established for drywall systems, where a metallic framework serves as the primary mechanism for load distribution, while gypsum or wooden oriented strand boards stabilize the framework.

This approach is in line with the increasing stringency of energy saving regulations in the construction sector, which are putting significant pressure on manufacturers of exterior concrete wall elements. These regulations require the achievement of minimum thermal conductivity in order to produce competitive elements for exterior walls. Such requirements have pushed the application of the material to the limits of its intended use under regulatory conditions. Against this background, the new prefabricated construction method is intended to offer an alternative to satisfy the high market demand for low-cost, fast-to-build, and durable buildings. The low thermal conductivity of the building envelope made of optimized LAC allows for meeting such demands. It is a sustainable solution since it can be separated and recycled easily and completely due to its clear materiality. The new construction method is an innovative solution that optimizes the use of lightweight concrete and steel and combines them in a sustainable way. The benefits of this new wall element design include high thermal insulation, fire resistance, sound insulation, durability, and flexibility. The wall element can be customized to meet different architectural requirements and aesthetic preferences. The prefabrication process reduces labor costs and on-site errors, shortens construction time, and improves safety. The new construction method is suitable for use in multi-story residential buildings as well as industrial or commercial buildings. The construction system could also be used for adding additional floors to buildings. Similar to the currently common construction designs, the low weight ensures comparatively low dead loads of the slabs.

### 1.1. Background Information on Lightweight Aggregate Concrete (LAC)

The utilization of stationary production methods for concrete components facilitates the standardization and mass production of prefabricated elements, thereby playing a critical role in the industrialization of the construction process [3,4,5]. The development of standardized elements goes along with the steadily advancing rationalization of the construction process and promises competitive advantages over cast in place concrete construction due to shorter construction times, reduced labor costs, weather-independent production, and high quality assurance [3,4,5]. Typical applications of the precast construction method in building construction include industrial buildings and warehouses, parking garages, office buildings, hospitals, schools, and multistory residential buildings [3,4,5]. In structural engineering, precast elements are used in particular when a significant cost advantage can be expected or when the construction conditions at the site cannot be achieved otherwise at a justifiable cost [4]. This applies, for example, to the manufacture of bridges, towers, or masts. Other important product areas include elements for water and wastewater management (e.g., tanks, pipelines, or sewage systems), and elements for traffic infrastructures (e.g., railroad ties, noise barriers, or modular paving) [3,4,5].

In the course of the 1990s, national standards and regulations were technically revised so that the use of lightweight concrete was standardized on an equal level with normal concrete [6]. Structural lightweight concrete (LC) suitable for reinforced concrete with a closed structure is regulated in EN 1992-1-1 [7] and EN 206 [8]. In addition, lightweight aggregate concrete (LAC) with an open structure for prefabricated reinforced components is covered by EN 1520 [1]. LAC is characterized by voids between aggregates formed by the omission or reduction of individual grain sizes that remain in the structure after compaction. The volume of the cement paste is reduced to such a degree that the aggregates are just pointwise interconnected [9,10]. LAC is produced almost exclusively with coarse lightweight aggregates, which is why the term no-fines is also used synonymously, although low-fines would be more suitable [10]. From the point of view of concrete technology, the structure of the LAC thus differs significantly from that of the dense structural LC. LAC is used to produce wall blocks and load-bearing and insulating wall elements, as well as non-structural components such as noise barriers or drainage concretes [11]. A new market segment opened for LAC wall elements due to the concrete technology available today (e.g., concrete admixtures and agents, as well as the continuous development of manufacturing processes and machine technology), along with the availability of lightweight aggregates (LWA) with particularly low density (e.g., expanded glass). This development allowed for low-density exterior walls to achieve thermal conductivity without the need for additional insulation.

### 1.2. Research Significance

Despite the widespread use of LAC in various construction applications, the innovative approach lies in its integration with the steel framework to create an energy efficient building envelope. The building envelope requires no additional insulation materials and provides thermal performance that is at least competitive with traditional envelope systems. This unique combination addresses key challenges in the construction industry to reduce energy consumption and improve building performance. This research gap highlights the need to evaluate the potential and suitability of this approach. This study will address the following three key research questions:

1. Feasibility of manufacturing an optimized LAC below the normative limits: A key challenge for manufacturers of precast monolithic walls is to achieve significantly lower thermal conductivity than conventional construction techniques, due to more stringent thermal insulation requirements. The purpose of this question is therefore to examine the feasibility of using a LAC+ with the greatest possible reduction in density and subsequent thermal conductivity while maintaining sufficient strength as a component in a new prefabricated wall system.
2. Assessing the robustness of LAC+ stability across varied conditions: The stability of the LAC+ must be maintained even during time-dependent deformations (e.g., shrinkage and creep) or under continuous loading. In addition, the compatibility of the LAC+ with the conventional compaction techniques used in the precast industry (roller compaction and/or vibratory compaction) should be verified, taking into account the change in consistency caused by the foam and the obstacles created by the steel profiles. This assessment is essential to ensure the quality and structural integrity of the precast elements even under full-scale conditions.
3. LAC+ as structural reinforcement: In this study, LAC+ acts as a critical element in the structural reinforcement, providing lateral bracing to the steel framework. By encasing the steel profiles, LAC+ provides stability and effectively prevents buckling of the integrated steel framework. As a result, a novel testing methodology is used to assess the overall strength and stability of the structure.

To address these research questions, a comprehensive approach was taken. Given the novelty of the LAC+ material technology at the anticipated low density, no previous studies have examined its fundamental material characteristics. Consequently, extrapolating existing standards becomes uncertain. Hence, an experimental investigation was conducted to evaluate the characteristic properties of LAC+. Therefore, three real-scale batches were produced in two precast plants as full-scale trials. Additionally, representative composite specimens were utilized at a smaller scale to demonstrate the capacity of LAC+ in stabilizing the load-bearing sheet steel profiles. Furthermore, prototypes were manufactured as a proof of concept of the potential and suitability of this approach for practical applications. These prototypes consist of full-size wall sections with connection details such as exterior wall joint, transport anchors, and a window opening. The prototypes were manufactured in both partners’ precast plants to ensure consistency and reliability.

By implementing this comprehensive approach, this study provides empirical data, practical validation, and prototypes that collectively support the potential application of the new construction method for building envelopes and defines a practical testing methodology to address the unique challenges.

## 2. Materials and Methods

### 2.1. Materials

#### 2.1.1. Sheet Steel Profile

In general, different sheet steel profile geometries can be considered for the framework. For the present project, a Sigma-profile was selected as a suitable candidate for the vertical steel profiles of the framework. This profile is commercially available in a wide range of dimensions. A relatively small dimension was chosen to demonstrate suitability in the lower load range and to limit the required dimensions for the later composite specimens (e.g., concrete cover or required slenderness for buckling). The Sigma profiles are cold-formed symmetrical profiles with parallel flanges and lips. The parallel flanges facilitate easy connection with other elements via welds or bolts, which is particularly useful when used in combination with the U-channel profiles within this project. The lips increase its torsional stiffness and stability. Sigma profiles have high load-bearing capacities and are already used in industrial and non-industrial steel constructions such as warehouses, hangars, and barns [12]. They provide material savings and cost advantages compared to heavy steel profiles due to their low weight, flexibility in the manufacturing process, and easy assembly [12]. They also have good corrosion resistance, which is essential for the present study given the high porosity of the LAC+ used. Additionally, they can be perforated and coated according to the project requirements. Table 1 summarizes the relevant technical parameters for the thin-walled sheet steel profile used.

The profiles are manufactured in accordance with EN 1090-2 [13] in a continuous roll forming process. The raw material of the profiles is a steel strip of grade S320GD according to EN 10346 [14].

#### 2.1.2. LAC+ Raw Material Properties and Mix Design

For the two precast plants involved, the mix design provided in Table 2 was used for the test program. A CEM I 42.5 R (precast plant 1) and a CEM I 52.5 R (precast plant 2) serve as binders. As LWA, expanded clay (Liapor F2.9 E) manufactured by Liapor GmbH & Co. KG (Hallerndorf-Pautzfeld, Germany) and expanded glass (Liaver 2–4 mm) manufactured by Liaver GmbH & Co KG (Ilmenau, Germany) are used in conforming with EN 13055-1 [15], and their physical properties are summarize in Table 3.

In the first step of the mixing process, the LWA is mixed with the specified amount of saturation water and part of the required mixing water. After pre-mixing, the binder, the powdered admixtures, and the additives are added. After a brief homogenization of the components, the remaining mixing water is added. When adding liquid admixtures, care must be taken to ensure that dry LWA in particular does not absorb the admixtures, which would result in reduced effectiveness of the admixtures. Liquid admixtures should therefore be added as the last component. Alternatively, they should be mixed with the remaining mixing water beforehand. After all components have been added, the minimum mixing time should be at least 60 s, depending on the mixing intensity, in order to obtain a uniformly mixed fresh concrete. Finally, the foam is added and mixed for a short period of about 30 s. The mixing process is summarized in Table 4. The mixing process described is an established method for producing lightweight concrete. While there may be different approaches that give similar results, the suitability of each method depends on specific conditions such as the capabilities of the production plant or the saturation options for LWA. For more general information on the production of lightweight concrete, refs. [9,11,16] provide additional insight.

#### 2.1.3. Manufacturing Process and Porosification

Both precast producers use compulsory mixers with additional agitators to homogenize the fresh concrete. At precast manufacturer 1, the LWA is stored in open boxes and exposed to the weather in the outdoor area of the plant, while at precast manufacturer 2, the LWA is stored in covered silos. Irrespective of the storage situation, both manufacturers must take into account the water absorption of the LWA in their mix design. Another difference between the two manufacturers is the compaction method of the fresh concrete. Whereas precast manufacturer 2 uses vibrating roller compacting, precast manufacturer 1 uses the vibrating or shaking technique for compacting the elements. The compaction technique installed is decisive to the extent of the porosification process of the cement matrix.

Based on preliminary tests on a laboratory scale, various LAC+ formulations were developed and further optimized for the respective production-specific boundary conditions. Balancing the trade-offs between density, strength, and thermal conductivity in LAC+ involved a systematic process of mix design optimization. The first step was to establish a minimum compressive strength requirement of approximately 1 N/mm^2^ to ensure sample stability during curing, as this strength threshold was considered sufficient for safe handling and processing within the context of the project and the capabilities of the manufacturers. Once this critical strength parameter was established, the focus shifted to reducing the density of the concrete. The primary approach used was to porosify the binder paste, which significantly improved the thermal insulation properties of LAC+. The exceptionally low density of LAC+ is achieved by introducing fine pores into the binder paste. The fine pores are created with a foam generator using air, water, and foaming agents. Foam is generated by applying air pressure to a mixture of foaming agent and water in the foam generator. The control of the applied pressure of air and water, as well as the dosage of the foaming agent, defines the properties of the foam produced. The pore structure itself, as well as the shape and distribution of the air bubbles within, significantly determines the properties obtained. In accordance with the desired performance of the concrete, varying proportions and different LWA types were used. In order to achieve the desired material properties, it was first necessary to find a suitable ratio between water and cement, as well as between foam, cement paste, and LWA. This optimization process was carried out to meet stringent energy efficient construction standards whilst ensuring structural stability, resulting in a refined mix design that sets a new standard for competitive lightweight aggregate concrete solutions.

**Figure 2:**
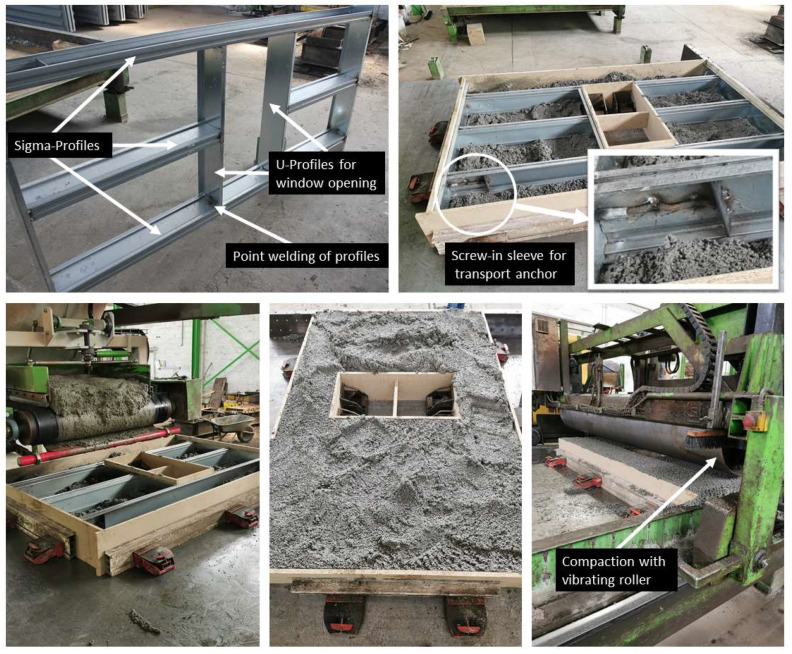
Manufacturing process of prototype at precast plant. (**Top left**) framework of steel profiles, rotated by 90°; (**Top right**) Partial filling of the formwork with LAC+; (**Bottom**) Filling and compacting of the wall element.

An impression of the manufacturing procedures in the precast plant is given in Figure 2. On the top left side, the framework of steel profiles can be seen, which is rotated later by 90° and placed in a test mold (Figure 2, top right). The interruption of the sigma profile in the middle part serves as a breakthrough and represents a window opening in the later wall system. The adaptation of the framework can be carried out at this point with the same U-profiles used in the framework for the upper and lower closure of the Sigma-profiles.

### 2.2. Experimental Program

Samples for three more comprehensive test series were produced in the precast plants to characterize the LAC+, as well as the construction system. First, two test series (Series 1 and 2) were produced in each precast plant. Based on the results of the first two series of tests, another series of test specimens (Series 3) was fabricated. Cores were drilled from elements for each series of tests in order to characterize the LAC+ as defined in [1]. The cores have a diameter of 150 mm and a height of 300 mm. They were cut to length and ground plane-parallel before the respective tests.

#### 2.2.1. Mechanical Properties

The compressive strength of LAC+ was determined after 28 days on drill cores (h/d = 300/150 mm) according to EN 1354 [17]. The modulus of elasticity was determined according to EN 1352 [18] on drill cores (h/d = 300/150 mm) after 28 days.

#### 2.2.2. Shrinkage and Creep

Shrinkage and creep deformations were determined on drill cores according to DAfStb booklet 422 [19] in a climate chamber at 20 °C and 65% rel. humidity. For measuring the shrinkage deformation, displacement gauges were attached to three drill cores at a distance of 200 mm on opposite sides and the changes in length were measured at fixed times up to an age of 460 days.

Three drill cores were loaded with a constant creep stress and the deformation measured and recorded for each core using three displacement gauges. The applied creep stress is one third of the average compressive strength after 28 days. The measurements of shrinkage and creep were started at a concrete age of 30 days. The average total deformation of the cores was recorded at specified times up to an age of 460 days.

#### 2.2.3. Dry Density and Thermal Conductivity

The dry density was determined according to EN 992 [20]. Thermal conductivity was measured by means of the transient plane source method (Hot Disk TPS 2200, Gothenburg, Sweden) according to EN ISO 22007-2 [21]. Prior to measurement, the samples were dried to constant mass at 105 °C and then cooled to a temperature of 23 °C. During cooling, the samples were kept in an airtight container filled with silica gel to ensure dry conditions. The thermal conductivity was determined on three different specimens and on different specimen sides in each case.

#### 2.2.4. Coefficient for Long-Term Effects on Compressive Strength

From a practical construction point of view, concrete is subjected to compressive and tensile forces in various structural applications, which can take the form of monotonic, cyclic, or sustained loading [22,23,24,25]. Failure of normal concrete is initiated by structural changes in the microstructure, such as cracking [24,25,26,27,28]. However, the characteristics of these structural changes depend on a variety of boundary conditions such as loading rate, water content of the concrete, specimen geometry, or type and direction of loading [25]. Knowledge of the stress-strain relationship and fracture mechanisms is crucial for predicting the behavior of concrete under different loading conditions [22,24,25,26,27,28,29]. While Rüsch [26,27] carried out important groundwork more than 60 years ago, extensive research has since led to the development of numerous models aimed at predicting stress–strain relationships and also failure states of concrete due to long-term effects. One topic in this context is the study of concrete under sustained loading. In such cases, a critical threshold can be defined as the ratio of the applied compressive stress over a given time period to the reference compressive strength. Once a critical threshold is exceeded, stress-induced structural changes lead to unstable damage progression [23,25,26,27,30]. A large number of research projects have attempted to establish a relationship between stress level thresholds, age under load, and duration of exposure, using different experimental programs. A comprehensive review of the different considerations and methodologies is provided by [23], where the authors compared six different scientific studies and programs in terms of reference strength, observed threshold, age of testing, duration of testing, and failure under sustained loading. In addition, they proposed a new method for predicting the failure of concrete under different long-term loading patterns based on the results of their experimental program. Another good review of the different experimental programs with respect to different types of concrete can be found in [25].

Although it has not been possible to derive a uniform experimental program nor a fixed limit, values between 0.70 and 0.90 have generally been determined for the critical stress level thresholds. Building codes, such as Eurocode 2 [7] and Model Code 2010 [31], address this issue through reduction coefficients. For structural lightweight concrete, this reduction coefficient α_cc_ is given in the National Annex (Germany) of Eurocode 2 [7] as 0.85. DIN EN 1520 [1] also recommends a reduction factor of 0.85 for LAC.

It is essential to note that the LAC+ investigated in this project possesses properties that lie outside the scope of these standards, necessitating the demonstration of the validity of these material–technological correlations. Since long-term experimental testing was impractical due to the extended testing duration, a short-term testing method was employed.

Specifically, drill cores were first stored in a standard climate (20 °C, 65% rel. humidity) until an equilibrium moisture content was reached. At 91 days of concrete age, the conditioned test specimens were loaded at a low loading rate (1 N/s) until fracture, meaning that the test specimens were subjected to a steadily increasing continuous load over a duration of approximately 6–7 h per specimen. During the test, inductive displacement transducers in the load direction recorded the deformation. The coefficient for long-term effects on compressive strength is determined by the quotient of the ultimate load achieved in the test and the average compressive strength of the drill core. The tests were carried out representatively for the LAC+ on six cores of Series 3. The test methodology is known from the literature and has been used, for example, to evaluate the sustained load of aerated concrete and normal concrete [24,25]. A similar test program was also carried out on infra-lightweight concrete [32,33], which has some similarities to LAC+ in terms of material technology.

#### 2.2.5. Trials on Composite Specimens

Small-scale composite specimens were designed and fabricated to investigate and demonstrate the stabilizing effect of the LAC+ on the steel sheet profiles in a compression test. The compressive load capacity of the composite specimens is compared with the test results of identical plain steel sheet profiles in a further step.

The vertical sigma profiles are decisive in this context. Due to the slender profile geometry, these profiles can buckle if a certain length is exceeded before the maximum permissible compressive stress of the material is reached. This buckling can be observed already for the specimen lengths tested here. Thus, the vertical sigma profiles need stabilization. The U-profiles at the upper and lower ends are mainly used for load transfer and for easier fixing in the formwork. The composite specimens have a height of 580 mm and a cross-section of approximately 250 × 250 mm^2^, which corresponds to a concrete cover of 37.5 mm on the wide side of the Sigma-profile. The sheet steel frames were fixed in specially manufactured formwork and concreted with LAC+ (Series 3) at the precast manufacturer.

## 3. Results and Discussion

### 3.1. Hardened Concrete Properties

In general, the optimum compromise between density, strength, and thermal conductivity is at the forefront of LAC+ mix design. Based on the preliminary laboratory tests, the desired material properties are achieved through the systematic use of chemical additives and admixtures, together with adjustments to the material composition and manufacturing process. As the complexity and performance of the material increases, so does the sensitivity of the material’s behavior. The type and content of the cement and aggregates, the characteristics of the cementitious foam, the type and concentration of the additives and admixtures, and the timing of their addition have been identified as the main influencing factors. Based on results of initial trials, three larger test series were produced in the precast plants. Table 5 provides the parameters of the LAC+ for the test series produced in the two precast plants.

**Figure 3:**
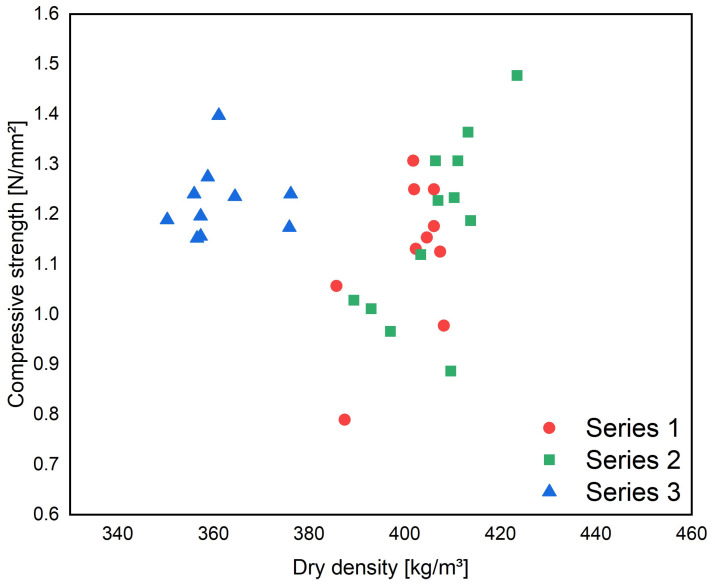
Compressive strength after 28 days for test Series 1, 2, and 3 as a function of the dry density.

Figure 3 illustrates the relationship between the compressive strength and dry density of the three test series.

The dry densities achieved were around 400 kg/m^3^ for the first two test series. The scatter of strength values was significant, reflecting the challenges faced by the precast plants mentioned earlier. By further optimizing the manufacturing process, the dry density was reduced to 360 kg/m^3^ in test Series 3. The variations in dry density are relatively small for all series. The difference between the heaviest and lightest sample is 34, 58, and 22 kg/m^3^ for Series 1, 2, and 3, respectively. This fact indicates that the mix design can produce homogeneous and stable concrete even on a factory scale. This was accomplished for the strength values in Series 3 as well. Based on the preliminary laboratory tests, this was initially one of the major uncertainties in mix design.

**Figure 4:**
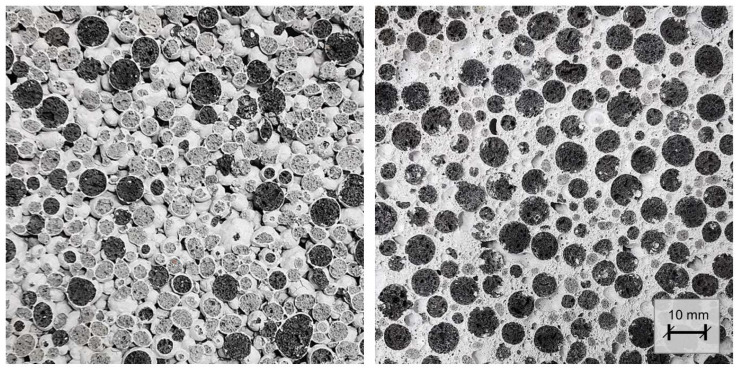
Series 3 (**right**) demonstrates significantly higher porosity compared to Series 2 (**left**), while Series 2 closely resembles typical lightweight aggregate concrete (LAC) in terms of its open-pore structure.

This uncertainty is due, on the one hand, to the specific nature of the LAC mix design and, on the other hand, to the degree of porosification of the paste between the LWA. With regard to the specific nature of LAC, it should be noted that the optimum water/binder ratio is only within a narrow range. Depending on the chosen LWA composition, a too high water to binder ratio will result in the cement paste draining from the surface of the LWA during compaction, while a too low water to binder ratio may result in adhesive agglomeration of the LWA [9]. With regard to the porosification of the paste between the LWA, it is essential that the foam maintains its quality and quantity in a stable manner. The additives used to produce the foam are usually very sensitive to variations in dosage, temperature, and mixing time. This becomes obvious, for example, in the pore structure of the artificial air pores. Other studies have shown that as the density of the foam decreases and mixing time increases, larger pore diameters are formed which tend to collapse during compaction [10] or result in reduced compressive strength of the hardened concrete [34]. Another special consideration when porosifying the paste is that only the voids between the LWA are filled. While too little foam will only result in inadequate filling of the voids between the LWA, too much foam would cause the LWA to simply float in the foam, and the load transfer along the LWA, which is characteristic of LAC, would no longer be ensured. This effect can be seen in Figure 4. While the left cross-section shows only partial porosification of the matrix (structure very close to conventional no-fines LAC), the right cross-section shows a correct and complete porosification of the paste filling the voids between the LWA.

**Figure 5:**
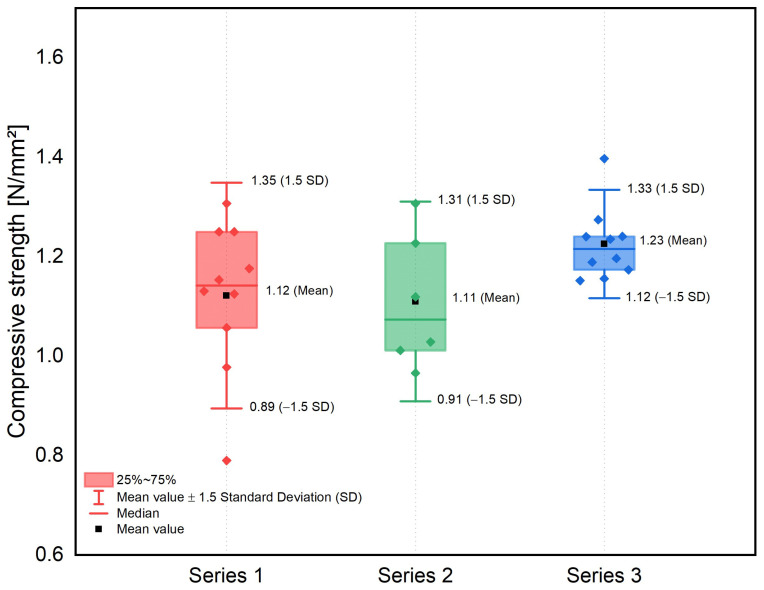
Compressive strength after 28 days of the LAC+ series with consideration of the statistical variations.

This fine tuning of the porosification determines the density, which is directly linked to the thermal conductivity, as well as the compressive strength of the LAC+, and thus the structural design of the components. In this context, a significant goal of the optimization of the LAC+ is achieving the lowest possible density and thus thermal conductivity. However, a reduction in these parameters is accompanied by a reduction in compressive strength. Further, the strength of the LAC+ must be sufficient to adequately stabilize the steel sections of the structure. A detailed overview of the compressive strength achieved for the optimized test series produced in the precast plants can be found in Figure 5.

The test results yield a compressive strength of the LAC+ of approx. 1.1 N/mm^2^. Furthermore, the statistical indicators for the quality control of concrete production were found to lie within the usual scatter range for LAC and confirm the previously formulated assumption of successful adjustment of the porosification of the paste between the aggregates. Series 3 exhibits a slightly higher compressive strength although its dry density is approximately 40 kg/m^3^ lower than the other series. Series 3 is an improvement of Series 1 and the higher compressive strength is probably due to a better match between foam and fresh concrete in the mix design (cf. Figure 5) and training of the personnel involved in the precast plant.

The porosification and volume of the hardened cement paste also affect the modulus of elasticity. According to the standard (DIN EN 1520 [1]), the modulus of elasticity can be estimated empirically from the dry density and characteristic compressive strength. For the present test series 1, 2, and 3, the estimated moduli of elasticity are 1016 N/mm^2^, 1106 N/mm^2^, and 1240 N/mm^2^, respectively. The experimentally determined moduli of elasticity (cf. Table 3) are consistently lower than the estimated values. This discrepancy can be attributed, in part, to the porosity of the hardened cement paste, as the reduced stiffness of the cement paste can lead to decreased resistance to deformation. It is important to consider the significant variability in the stiffness of different LWAs as well. In this context, it should be noted that the normative and empirical determination of the modulus of elasticity applies to all LWA approved by this standard. Therefore, the estimation reflects an average relationship and does not account for the specific characteristics of individual LWAs.

### 3.2. Compressive Strength Capacity under Sustained Loading

Strength under sustained compressive loading is an important parameter for the design and performance of LAC+. It reflects the ability of the material to resist deformation and prevent cracking under long-term service conditions. In general, the compressive strength capacity under sustained loading can be understood as an elementary microstructural threshold above which irreversible and unstable damage progression must be expected. For safe material utilization and saving of resources, a realistic coefficient for long-term effects on compressive strength must therefore be determined for the LAC+.

**Figure 6:**
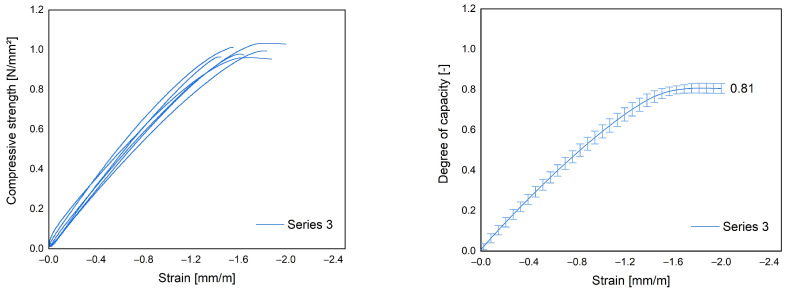
(**Right**) Relative load capacity (σ_sustained_/σ_28days_) of the compressive strength under sustained loading. (**Left**) Corresponding stress–strain curves for test Series 3 under sustained loading.

Figure 6 illustrates the variation in mean relative load capacity, defined as the ratio of the sustained compressive strength to the average compressive strength at 28 days, with respect to longitudinal deformations. The corresponding individual stress–strain curves are provided individually on the left.

The experimentally determined threshold for sustained loading is 81% for test series 3. This value is comparable to the known α_cc_ for long-term effects on compressive strength for lightweight concrete (according to Eurocode 2: 85% [7], cf. Section 2.2.4) and the one for LAC (85%) according to the DIN EN 1520 [1]. The normative and generalized determination of the reduction factor α_cc_ would overestimate the long-term performance of LAC+. This is confirmed by research conducted by Empelmann [35], who concluded that α_cc_ does not always provide conservative results. Furthermore, the relationship between longitudinal deformation and applied stress is very similar in the lower stress range and only deviates more strongly at higher compressive stresses. This behavior is known from concretes and speaks for a homogeneous load transfer of the optimized LAC+.

### 3.3. Shrinkage and Creep of the LAC+

**Figure 7:**
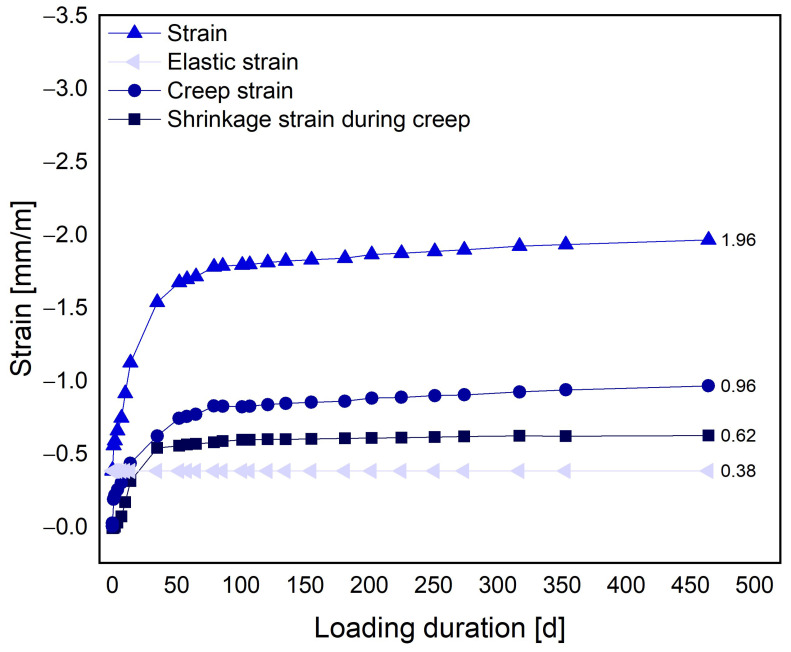
Strains during shrinkage and creep for test Series 2.

Time-dependent deformations can occur due to load-independent processes such as shrinkage or load-dependent processes such as creep [36]. Minimizing the deformations of the LAC+ is essential to minimize cracking, to restrain stresses, and to ensure a permanent bond between the LAC+ and the steel framework. Unlike normal concrete, the time-dependent deformations of LAC+ are largely determined by the aggregate used, although they do not show time-dependent behavior themselves [37]. The main influencing parameters of the LWA are their low density and the associated modulus of elasticity as well as their porosity and stage of water saturation. There is not a wide range of published empirical values for the deformation properties of LAC with a porosified matrix, especially for the present density ranges. Thienel [10] found that the shrinkage values of a LAC without fines and a properly manufactured LAC with a porosified matrix are similar, and at a density of 600 kg/m^3^ are approximately 0.6 mm/m. Thienel [10] also observed that a LAC with a porous matrix exhibited higher specific creep values as compared to a conventional LAC. However, the risk of shrinkage cracking was reduced due to the high relaxation potential of the LAC. DIN EN 1520 [1] suggests using 0.75 mm/m for drying shrinkage in an environment with 45 ± 5% rel. humidity. Figure 7 shows the shrinkage and creep deformation curves for test Series 2 over time.

The shrinkage deformation curves in Figure 7 are comparable with normal LAC. The results determined within the scope of the present tests are thus within the expected results. However, it must be noted in this comparison that the age of removal differs from the one set in [38]. In relation to the residual stress resulting from shrinkage deformation in precast concrete elements, it should also be noted that a significant portion of the deformation can be alleviated during the curing process in the precast plant and thus before installation on site.

### 3.4. Trials on Composite Specimens

**Figure 8:**
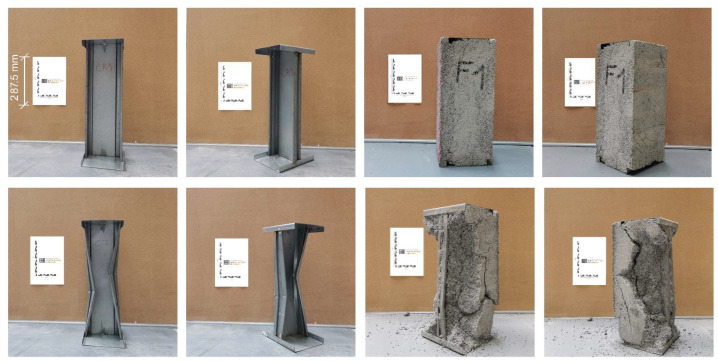
Comparison of test specimens for the composite system. On the left: plain steel profiles connected to horizontal U-profiles at the top and bottom by spot welding. On the right: identical steel profiles embedded in the LAC+. Compression tests for sheet steel profiles (**left**) versus composite specimens (**right**) before (**top**) and after (**bottom**) the test.

The composite specimens consist of a sheet steel framework embedded in the LAC+. The successful integration of the sheet steel framework into the LAC+ is critical to the overall development of the wall component. The framework of sheet steel profiles fulfils the load-bearing tasks of the component in the wall. The surrounding LAC+ prevents buckling of the steel profiles and thus yields sufficient load-bearing capacity. As there are no standardized test procedures for this new precast construction method, an individual test program was developed for the composite components (cf. Chapter 2.3). For this purpose, the load-bearing behavior of a plain steel profile is compared with that of an identical steel profile embedded in the LAC+. Figure 8 shows the specimens in comparison. The left half shows the plain steel profile, while the right half shows the composite specimen. The buckling failure of the plain steel frame is obvious. In contrast, the embedded steel frame exhibits no such deformation. Here, the failure was caused by spalling of the LAC+ which subsequently destabilized the steel frame and led to failure. The upper half of the photos shows the specimens before the test, while the lower half shows the specimens after the test.

**Figure 9:**
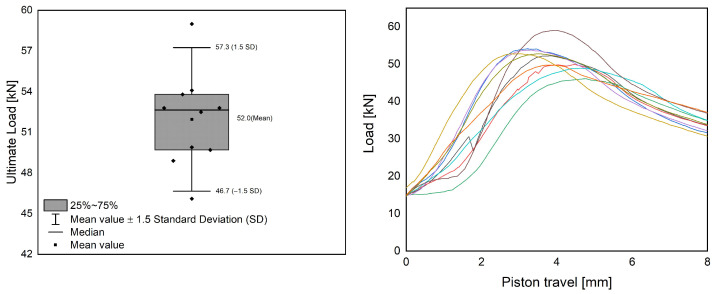
Box–Whisker diagram for compression test of sheet steel profile (**left**) with corresponding load-displacement diagrams (**right**).

In Figure 9, the results of the compression tests are summarized for the plain steel frames in a Box–Whisker diagram on the left side, while the corresponding load-displacement diagrams are shown on the right side.

**Figure 10:**
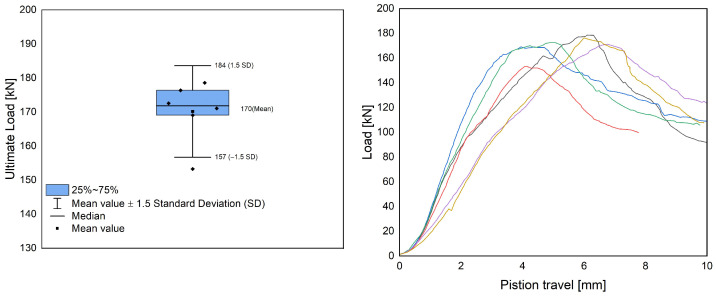
Box–Whisker diagram for compression test of composite specimens with LAC+ from Series 3 (**left**) with corresponding load-displacement diagrams (**right**).

The results of the compression tests on the sheet steel profiles confirm that the designed specimen type is suitable for the chosen test setup. The results in the compression test are reproducible and show only minor deviations from the mean value. The average ultimate load of the plain steel profiles is 52 kN and thus well below the value that can theoretically be achieved by material failure. Otherwise, buckling values of around 110 kN would have been expected (cf. Table 1). Thus, the test setup successfully triggers a stability failure of the profile. Figure 10 exhibits the results of the compression tests for the composite specimens.

The results of the experimental investigation show that the use of LAC+ as an embedding material for steel sheet profiles can significantly improve their load-bearing capacity. The composite specimens exhibited an ultimate load of 170 kN, which is more than three times that of the plain steel sheet profile (52 kN) and more than 50% higher than the maximum compressive load capacity specified by the manufacturer (110 kN, cf. Table 1). This indicates that the bond between the sheet steel profile and the LAC+ was effective in transferring the load from the sheet steel profile to the concrete section and preventing stability failure due to buckling. However, it should be noted that these results are based on a limited data set and do not allow for generalization or the derivation of design formulae to calculate the compressive capacity of composite specimens with different concrete qualities, profile geometries and types, and concrete coverings. Further research is needed to investigate these variables and to extend the test program to include strength tests on mock-up walls, which would better reflect the actual behavior of composite elements in structural applications. A possible reference for such a test program could be DIN EN 1520 Annex B (design of components based on tests) [1] or ASTM E72 [39], which provides standard methods for conducting strength tests on structural panels.

### 3.5. Implementing Laboratory Findings in Production: Demonstrator

In general, there are significant challenges in ensuring the applicability of laboratory tests to full-scale production in manufacturering plants. One notable difference is the controlled environment of laboratories, with optimum humidity and temperature conditions, and calibrated and more precisely controllable instruments (e.g., mixing energy and time). However, when testing on a larger scale, such as in production plants, there are unique considerations for lightweight concrete. These may include the need for specialized equipment, such as foam generators, to accommodate complex mix designs. In addition, factors such as saturation of LWA, which can be controlled precisely in the laboratory, need to be taken into account, whereas in concrete plants with open storage and weathering, greater variations are to be expected. In addition, the development of heat of hydration becomes more of an issue with larger samples, such as wall elements, where heat dissipation is less efficient compared to smaller laboratory samples.

**Figure 11:**
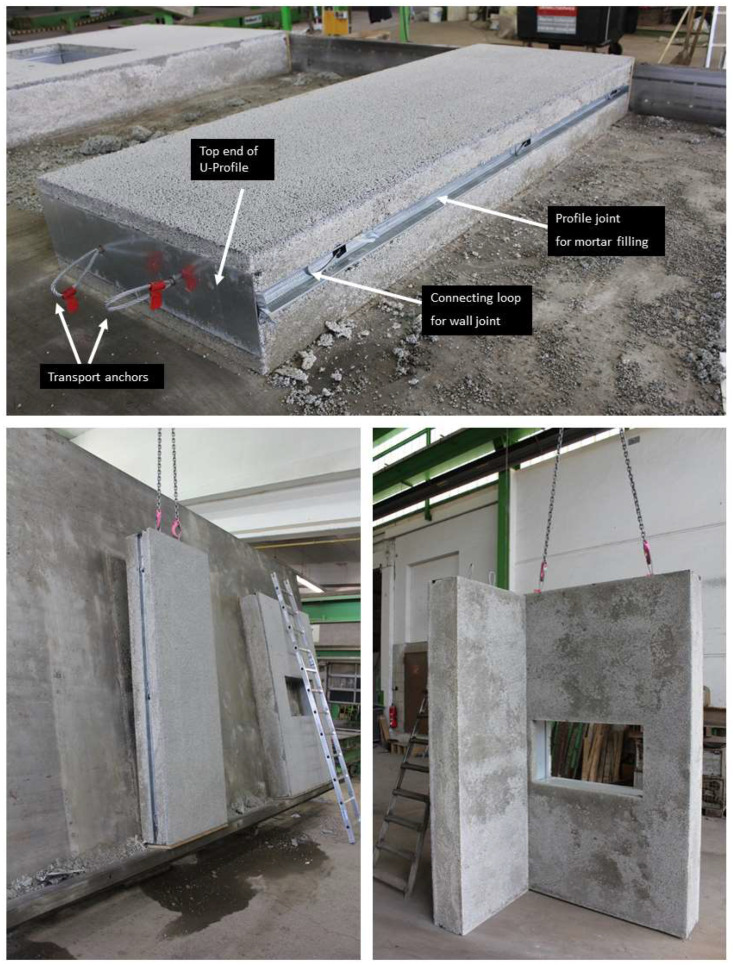
Connecting and anchoring elements for transporting, lifting, and connecting the individual precast elements.

In the context of the research presented, there are also a number of considerations that need to be taken into account when handling the samples, given the exceptionally low strength of the concrete. The performance of a structural component is influenced by various factors, including the composite effect between the LAC+ and the steel framework. However, the transfer of shear forces, as well as the introduction of local compressive and tensile forces, are crucial for the overall performance of the component. To introduce these local loads into the construction system, connection and anchorage elements are used. During the transportation, lifting, and assemblage of individual precast elements, there is a risk of introducing large and variable loads into the component. Hence, careful consideration must be given to the design and implementation of these elements to ensure the safe and efficient functioning of the component. The implementation and handling of the adapted connecting elements was tested exemplarily on the prototypes. Figure 11 gives an impression.

The feasibility of the proposed new construction method, through the successful translation of laboratory findings into production, offers an efficient use of building materials with improved energy efficiency in the building environment, striving towards sustainable building practices. As a result, the proposed building envelope does not require additional insulation materials and provides thermal performance that is at least competitive with traditional envelope systems.

## 4. Conclusions

This study aimed at presenting a novel construction method for prefabricated wall elements, in which a filigree steel framework takes over the load-bearing function, and a tailor-made LAC provides thermal insulation and stability. To accomplish this, the properties of LAC were optimized, with the objective of achieving the lowest possible density and thermal conductivity while maintaining sufficient strength to prevent framework stability failure. The optimized LAC+ was intended to create a building envelope that complies with current energy regulations without requiring additional insulation. The successful development of the novel construction method was validated in cooperation with two precast plants, demonstrating the transfer of laboratory findings to the real scale. The main findings can be summarized as follows:

1. The mechanical properties of the potential LAC+ candidates were fundamentally characterized on the basis of three extensive real-scale batches. A notable result of this characterization was the remarkable reduction in compressive strength to as low as 1.1 N/mm^2^. This reduction in compressive strength created a pathway for density reduction, ultimately achieving densities as low as 360 kg/m^3^. This in turn led to the achievement of competitive thermal conductivity values as low as 0.09 W/(m∙K). The porosification of the matrix between the LWA grains plays a special role in the successful transition from laboratory to real scale.
2. Short-term tests with a low loading rate revealed a reduction coefficient of 0.81 to account for the reduced compressive strength of the LAC+ under sustained loading. This result is comparable to the factor 0.85 proposed for LAC in DIN EN 1520 [1].
3. Composite specimens, which represent a wall section with a vertical sheet steel profile embedded in the LAC+, were tested to demonstrate the ability of the LAC+ to stabilize thin-walled sections against buckling. The mechanical performance of the framework was significantly increased with the use of LAC+.
4. Based on the obtained mechanical properties of the LAC+ and the composite tests, two prototypes were produced in precast plants of project partners. The design of the prototypes represents wall sections in original size and takes into account a connection detail (outer wall joint) and anchors (transport anchors), as well as a window opening. The experimental tests have thus been successfully transferred to the real scale.

For developing a comprehensive design concept for wall elements, it is necessary to conduct further tests that consider various profile types and cross-section geometries. Additionally, it is important to perform tests with various concrete covers and on the effective cross-section of the LAC+ within the wall element. These tests will provide insights into the behavior of the proposed construction method under different conditions and will support the development of an optimized design approach that meets the required performance criteria.

## Figures and Tables

**Table 1:** Manufacturer’s data for technical parameters of the sheet steel profiles used.

Parameter	Sigma Profile 175/61-1,0
Height [mm]	175
Width [mm]	61
Steel plate thickness [mm]	1.0
Cross-section area [mm^2^]	334
Section modulus [mm^3^]	18,280
Compressive load capacity [kN]	110
Shear force capacity [kN]	14.2
Bending moment capacity [kNm]	5.9

**Table 2:** Mix design composition of the LAC+ for the two precast plants for large-format production.

Material	Precast Plant 1 (Series 2 and 3)		Precast Plant 2 (Series 1)	
	Amount [kg]	Volume [dm^3^]	Amount [kg]	Volume [dm^3^]
Cement	120 kg	39	120	39
Water	96	96	85	85
Liapor F2.9 E (4/10 mm)	216	920	124	394
Liaver (2/4 mm)	21	109	126	642
Foam	10.8	197	8.65	350
Stabilizer (Sika ^1^ ST 3)	2	1.9	-	
Accelerator (Daraset ^2^ 304)	4	1.4	-	

^1^ Manufactured by Sika Deutschland GmbH (Stuttgart, Germany). ^2^ Manufactured by GCP Applied Technologies (Cambridge, MA, USA).

**Table 3:** Physical properties of the LWA used.

Aggregate Type	Shape	Crushing Resistance ^1^ [N/mm^2^]	Particle Density ^1^ [kg/m^3^]	Water Absorption ^1^ [wt.-%]
Liaver 2-4	Rounded	1.4	550	11.8
Liapor F2.9 E	Rounded	2.2	342	20.7

^1^ Manufacturers data.

**Table 4:** Mixing process of LAC+.

Step	Approx. Mixing Time [s]
Mixing of LWA with half of the water	60
Addition of cement and additives	90
Addition of remaining mixing water	60
Addition of foam	30

**Table 5:** Overview of the mechanical parameters for three test series of the optimized LAC+.

Parameter	Series 1 (Precast Plant 2)	Series 2 (Precast Plant 1)	Series 3 (Precast Plant 1)
Dry density [kg/m^3^]	400	407	358
Thermal conductivity [W/(m∙K)]	0.118	0.121	0.092
Mean compressive strength [N/mm^2^]	1.12	1.11	1.23
Characteristic compressive strength ^1^ [N/mm^2^]	0.93	0.86	1.15
Modulus of elasticity [N/mm^2^]	969	1046	878
Coefficient for long-term effects on compressive strength [-]	-	-	0.81
Shrinkage ^2^ [mm/m]	-	−0.62	-
Creep ^2^ [mm/m]	-	−0.96	-

^1^ According to EN 1520 [1]. ^2^ Measurement started 30 days after concreting.

## Data Availability

Data are contained within the article.
